# Variants of Streptococcus pneumoniae Serotype 14 from Papua New Guinea with the Potential to Be Mistyped and Escape Vaccine-Induced Protection

**DOI:** 10.1128/spectrum.01524-22

**Published:** 2022-07-12

**Authors:** Sam Manna, Leena Spry, Ashleigh Wee-Hee, Belinda D. Ortika, Laura K. Boelsen, Steven Batinovic, Nadia Mazarakis, Rebecca L. Ford, Stephanie W. Lo, Stephen D. Bentley, Fiona M. Russell, Christopher C. Blyth, William S. Pomat, Steve Petrovski, Jason Hinds, Paul V. Licciardi, Catherine Satzke

**Affiliations:** a Infection and Immunity, Murdoch Children’s Research Institute, Royal Children's Hospital, Parkville, Victoria, Australia; b Department of Paediatrics, The University of Melbourne, Parkville, Victoria, Australia; c Department of Microbiology and Immunology at the Peter Doherty Institute for Infection and Immunity, The University of Melbourne, Parkville, Victoria, Australia; d Department of Microbiology, Anatomy, Physiology, and Pharmacology, La Trobe University, Bundoora, Victoria, Australia; e Infection and Immunity Unit, Papua New Guinea Institute of Medical Research, Goroka, Eastern Highlands, Papua New Guinea; f Parasites and Microbes, Wellcome Sanger Institute, Hinxton, United Kingdom; g Wesfarmers Centre of Vaccines and Infectious Diseases, Telethon Kids Institute and School of Medicine, University of Western Australia, Perth, Australia; h Department of Infectious Diseases, Perth Children’s Hospital, Perth, Australia; i Department of Microbiology, PathWest Laboratory Medicine, Perth, Australia; j Institute for Infection and Immunity, St. George's, University of London, United Kingdom; k BUGS Bioscience, London Bioscience Innovation Centre, London, United Kingdom; Emory University School of Medicine

**Keywords:** serotype, capsule, pneumococcus, *Streptococcus pneumoniae*, variant, pneumococcal conjugate vaccine

## Abstract

Streptococcus pneumoniae (the pneumococcus) is a human pathogen of global importance, classified into serotypes based on the type of capsular polysaccharide produced. Serotyping of pneumococci is essential for disease surveillance and vaccine impact measurement. However, the accuracy of serotyping methods can be affected by previously undiscovered variants. Previous studies have identified variants of serotype 14, a highly invasive serotype included in all licensed vaccine formulations. However, the potential of these variants to influence serotyping accuracy and evade vaccine-induced protection has not been investigated. In this study, we screened 1,386 nasopharyngeal swabs from children hospitalized with acute respiratory infection in Papua New Guinea for pneumococci. Swabs containing pneumococci (*n* = 1,226) were serotyped by microarray to identify pneumococci with a divergent serotype 14 capsule locus. Three serotype 14 variants (‘14-like’) were isolated and characterized further. The serotyping results of these isolates using molecular methods varied depending on the method, with 3/3 typing as nontypeable (PneumoCaT), 3/3 typing as serotype 14 (seroBA), and 2/3 typing as serotype 14 (SeroCall and quantitative PCR). All three isolates were nontypeable by phenotypic methods (Quellung and latex agglutination), indicating the absence of capsule. Illumina and nanopore sequencing were employed to examine their capsule loci and revealed unique mutations. Lastly, when incubated with sera from vaccinated individuals, the 14-like isolates evaded serotype-specific opsonophagocytic killing. Our study highlights the need for phenotypic testing to validate serotyping data derived from molecular methods. The convergent evolution of capsule loss underscores the importance of studying pneumococcal population biology to monitor the emergence of pneumococci capable of vaccine escape, globally.

**IMPORTANCE** Pneumococcus is a pathogen of major public health importance. Current vaccines have limited valency, targeting a subset (up to 20) of the more than 100 capsule types (serotypes). Precise serotyping methods are therefore essential to avoid mistyping, which can reduce the accuracy of data used to inform decisions around vaccine introduction and/or maintenance of national vaccination programs. In this study, we examine a variant of serotype 14 (14-like), a virulent serotype present in all currently licensed vaccine formulations. Although these 14-like pneumococci no longer produce a serotype 14 capsule, widely used molecular methods can mistype them as serotype 14. Importantly, we show that 14-like pneumococci can evade opsonophagocytic killing mediated by vaccination. Despite the high accuracy of molecular methods for serotyping, our study reemphasizes their limitations. This is particularly relevant in situations where nonvaccine type pneumococci (e.g., the 14-likes in this study) could potentially be misidentified as a vaccine type (e.g., serotype 14).

## INTRODUCTION

Streptococcus pneumoniae (the pneumococcus) is a bacterial pathogen of global importance, as it causes a range of diseases, including pneumonia, sepsis, and meningitis (1). Pneumococci are also carried in the nasopharynx, which is typically an asymptomatic event but is important for transmission and progression to disease (2, 3). The capsular polysaccharide is a major virulence determinant and is the basis for pneumococcal classification. Over 100 pneumococcal serotypes have been described, but currently licensed pediatric vaccines only confer protection against a subset (up to 20) of these serotypes. Pneumococcal serotyping is essential for disease surveillance and vaccine impact. Molecular approaches used to infer the serotype have increased in popularity and rely on a preexisting database of reference capsule locus (*cps*) sequences (4). Unfortunately, there are limited genomic data from pneumococci from low and middle-income countries, particularly in the Asia-Pacific region. We have previously discovered new genetic variants from this region that can be ‘mistyped’ using common molecular methods (5). This reduces the accuracy of the serotyping data used to make decisions around vaccine introduction and around the monitoring of vaccine impact.

The burden of pneumococcal disease in Papua New Guinea (PNG) is among the highest, worldwide (6, 7). High-density pneumococcal carriage with a diverse range of serotypes occurs early in life (6, 7). In contrast with most other settings, randomized controlled trials in PNG showed pneumococcal vaccines have thus far had a limited impact on vaccine-type nasopharyngeal carriage (6, 7). Therefore, it is important to identify pneumococcal variants with the potential to evade vaccine-induced protection in PNG.

Serotype 14 is a highly invasive serotype (8) that is targeted by all currently licensed pneumococcal vaccines (PCV10, Pneumosil, PCV13, PCV20, and PPSV23). Variants of serotype 14 have been identified in Australia and South Africa. These variants are unable to be serotyped by phenotypic methods (‘nontypeable’) because mutations in the serotype 14 capsule locus mean that they no longer produce a capsule (9–11). However, an in-depth investigation of these variants has not been conducted. Molecular typing methods are now common, but the potential for mistyping these variants as serotype 14 via such methods is unclear. Additionally, the capacity of these variants to evade the antibody response elicited by vaccination has not been examined.

Here, we investigate variants of pneumococcal serotype 14 that we isolated from nasopharyngeal swabs in PNG. We report the source of the divergence and the phenotypic consequences on capsule production. Importantly, we investigate these variants for their potential to be mistyped by popular serotyping methods as well as whether they can evade vaccine-induced protection.

## RESULTS

We examined nasopharyngeal swabs from children hospitalized with acute respiratory infection as part of the PneuCAPTIVE study (12). We tested 1,386 swabs for pneumococci using *lytA* quantitative PCR (qPCR). Of these, 1,287 (92.86%) were positive (or equivocal) for the *lytA* gene. Following culture, α-hemolytic growth was obtained from 1,226 of the swabs, which were then serotyped via DNA microarray. Among these, three (0.24%) swabs contained a putative variant of serotype 14. These ‘14-likes’ comprised 3.29% of swabs where serotype 14 was detected (*n* = 91). A pneumococcal isolate from each of these samples was purified (PMP1437, PMP1438, and PMP1514). Each isolate was a distinct, novel genotype as defined by microarray arrayCGH, Multi-Locus Sequence Typing (MLST), and Global Pneumococcal Sequence Cluster (GPSC) (Table 1).

**TABLE 1:** Strain information of serotype 14 variant (14-like) pneumococci identified in this study

		MLST								
Isolate	Source	*aroE*	*gdh*	*gki*	*recP*	*spi*	*xpt*	*ddl*	Sequence type	GPSC
PMP1437	Nasopharynx of child with acute respiratory infection	7	5	792^*a*^	4	6	1	1	17329^*a*^	1000^*b*^
PMP1438	Nasopharynx of child with acute respiratory infection	1	5	4	8	9	277	1123^*a*^	17330^*a*^	999^*b*^
PMP1514	Nasopharynx of child with acute respiratory infection	1	5	793^*a*^	5	9	1	1	17331^*a*^	998^*b*^

a Novel allele and/or sequence type identified in this study.

b Novel GPSC type identified in this study.

**FIG 1:**
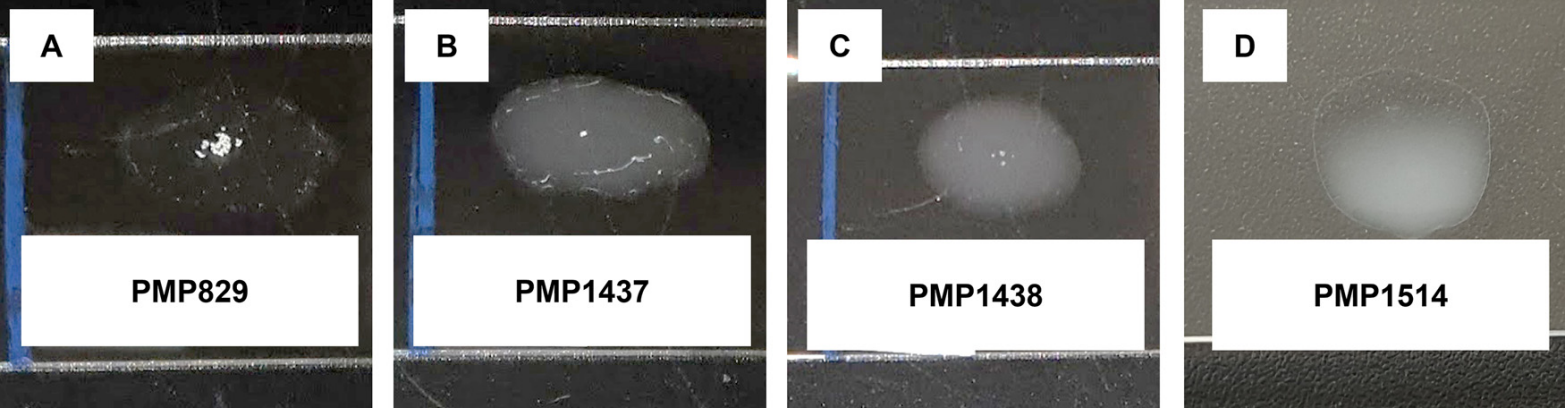
Representative latex agglutination reactions of a serotype 14 positive-control strain PMP829 (A) and 14-like isolates PMP1437 (B), PMP1438 (C), PMP1514 (D). Bacterial suspensions were mixed with a latex reagent that contained serotype 14 antibodies from type 14 antisera from Statens Serum Institut adsorbed to polystyrene latex beads. A positive reaction is indicated by visible agglutination and clearing of the suspension, whereas a negative reaction lacks agglutination and remains uniform and white/opaque.

**FIG 2:**
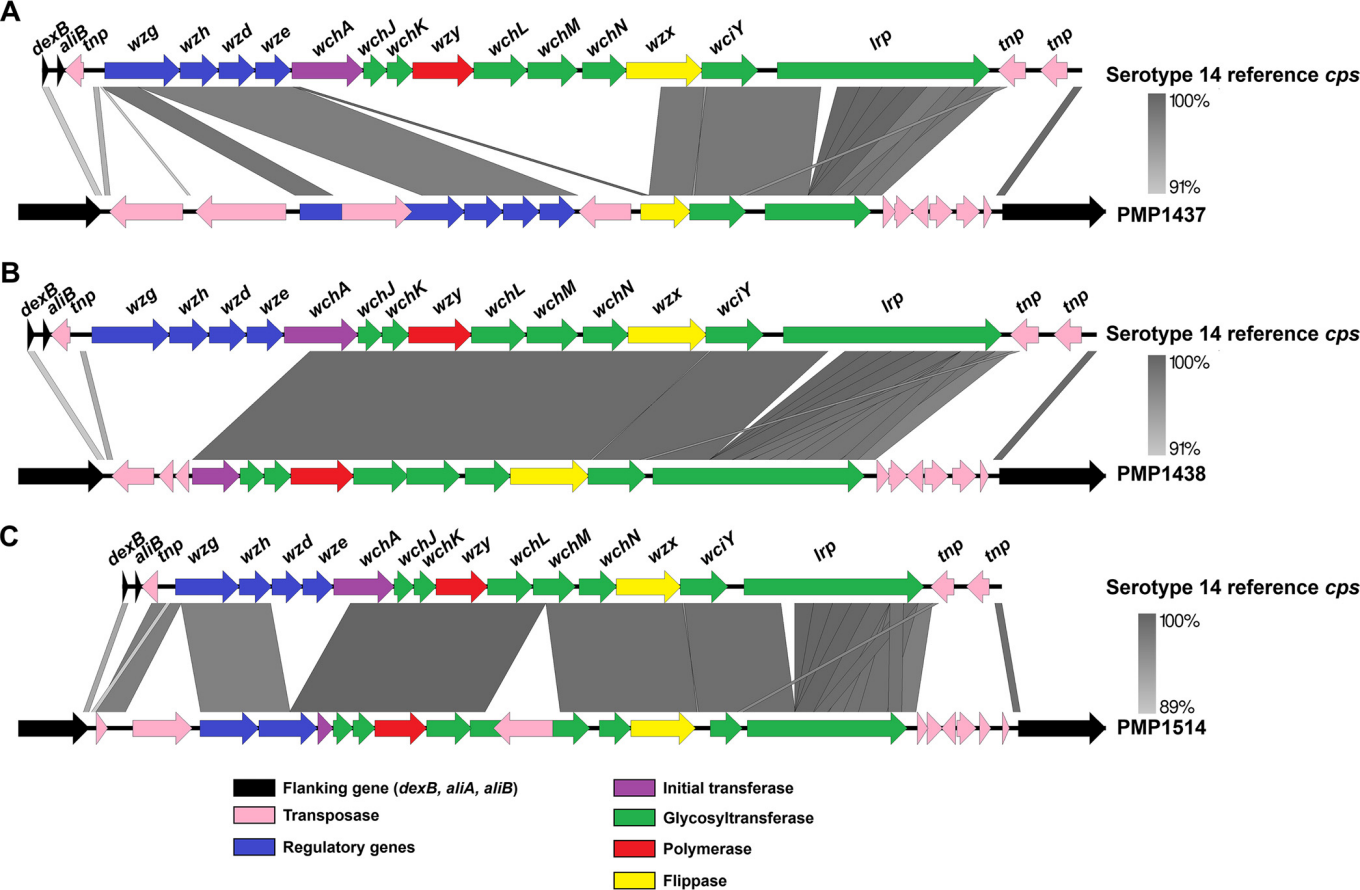
Comparison of the capsular polysaccharide loci of 14-like pneumococci PMP1437 (A), PMP1438 (B), and PMP1514 (C) to the reference serotype 14 sequence strain 34359 (4). Schematics of the sequence comparison were generated using Easyfig version 2.2.5 (30).

To determine if these 14-like pneumococci are ‘nontypeable’, similarly to those previously identified in Australia and South Africa (10, 11), we applied phenotypic serotyping methods. All three isolates were nontypeable using both Quellung and latex agglutination (Fig. 1 and Table 2). To verify the presence of a defective serotype 14 *cps* locus in these isolates, we performed whole-genome sequencing. The reads (Illumina and Nanopore) were combined, and the resultant assemblies for each isolate (GenBank accession: OM328061-OM328063) were used to interrogate the *cps* locus (4). Each 14-like isolate has mutations in their *cps* locus. PMP1437 has a 6.9 kb deletion, including *wchA*, *wchJ*, *wchK*, *wzy*, *wchL*, *wchM*, *wchN*, and part of *wzx* as well as a 1.3 kb insertion of an IS1380 element in the *wzg* gene. The *cps* locus in PMP1438 has a 4.2 kb deletion consisting of *wzg*, *wzh*, *wzd*, *wze*, and part of *wchA*. Lastly, PMP1514 has a 1.8 kb deletion, including *wzd*, *wze*, and part of *wchA* as well as disruption of the *wchM* gene with an IS1380 element (Fig. 2). These findings were consistent with the microarray results.

**TABLE 2:** Summary of serotyping results of serotype 14 variant (14-like) pneumococci from PNG

	Serotype result^*a*^						
	Molecular serotyping methods					Phenotypic serotyping methods^*e*^	
Isolate	Microarray^*b*^	PneumoCaT (% coverage)^*c*^	seroBA	SeroCall	Serotype 14 qPCR (ct value)^*d*^	Quellung	Latex agglutination
PMP1437	14-like	Nontypeable (44.50%)	14	Nontypeable	Negative (no ct)	Nontypeable	Nontypeable
PMP1438	14-like	Nontypeable (68.52%)	14	14	Positive (22.75)	Nontypeable	Nontypeable
PMP1514	14-like	Nontypeable (84.21%)	14	14	Positive (19.78)	Nontypeable	Nontypeable

a The number outside parentheses refers to the serotype call made by that method, e.g., ‘14’ indicates ‘serotype 14’.

b Serotype by DNA microarray was initially determined from a swab following a culture amplification step and was subsequently repeated on the isolates derived from these samples to validate that the 14-like strains were isolated.

c The number inside parentheses represents the percent coverage against the serotype 14 *cps* locus, which was the top *cps* locus match for all three isolates.

d The number in parentheses represents the mean cycle threshold (ct) value obtained by quantitative PCR (qPCR) (*wzy* gene target) from duplicate wells.

e Phenotypic serotyping was performed with all SSI pools and type 14 sera.

To determine the potential for the 14-like pneumococci to be mistyped, we applied four molecular methods: three whole-genome sequencing methods (PneumoCaT [13], seroBA [14], and SeroCall [15]) and singleplex qPCR using the CDC serotype 14-specific qPCR primers and probe (16). PneumoCaT made no serotype call (nontypeable), while seroBA designated these isolates as serotype 14 (Table 2). Interestingly, SeroCall and qPCR designated PMP1438 and PMP1514 as serotype 14, whereas PMP1437 was designated as nontypeable by these methods (Table 2).

**FIG 3:**
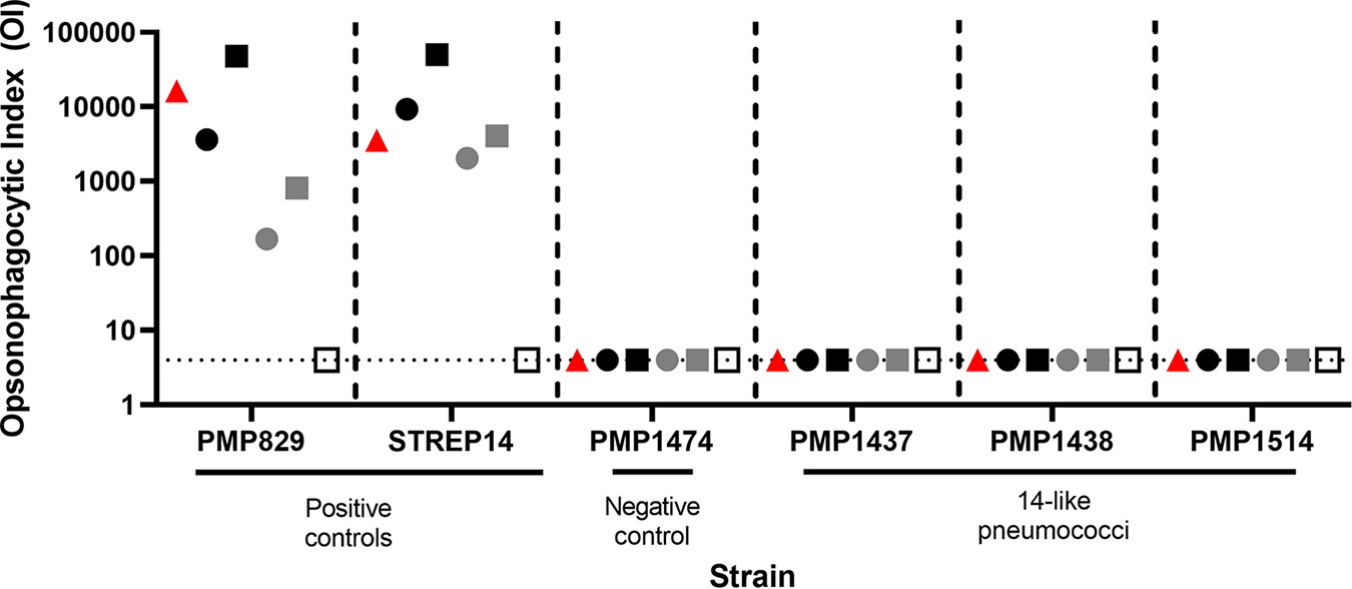
Opsonophagocytic indices of serotype 14 (PMP829, STREP14), nonencapsulated (PMP1474) and 14-like (PMP1437, PMP1438, and PMP1514) pneumococci. Isolates were incubated with either 007SP reference sera (red triangles) or sera from individuals vaccinated with PCV7 and/or PPSV23. Each symbol represents an individual serum sample with black, gray, and white colors representing serum samples with high (>10 μg/mL), medium (1 to 5 μg/mL), and low (<0.5 μg/mL) serotype 14 IgG titers, respectively. Times specified in brackets below are the ages of the children at the time of vaccination. Red triangle, 007SP reference sera; black circle, 1 dose PCV7 (2 years); black square, 1 dose PPSV23 (12 months) + 1 dose PCV7 (2 years); gray circle, 1 dose PCV7 (2 years); gray square, 2 doses PCV7 (14 weeks and 2 years); white square, 2 doses PCV7 (6 and 14 weeks) + 1 dose PPSV23 (12 months). OI 4 (dotted line) or less is defined as negative for opsonophagocytic killing. Nonspecific killing in this assay was −25%, −12%, −2%, 21%, 2%, and 20% for PMP829, STREP14, PMP1474, PMP1437, PMP1438, and PMP1514, respectively.

As the 14-like pneumococci appear to be unable to synthesize capsule, we hypothesized they would be able to escape opsonophagocytic killing mediated by the serotype 14 antibodies elicited by vaccination. To test this, we performed opsonophagocytic assays using sera (with a range of serotype 14-specific IgG values) from individuals vaccinated with PCV7 and/or PPSV23, both of which target serotype 14. All three 14-like isolates evaded serotype-specific opsonophagocytic killing, in contrast with the capsule producing serotype 14 control strains (PMP829 and STREP14) (Fig. 3).

## DISCUSSION

S. pneumoniae serotype 14 is one of the major capsule types associated with pneumonia and invasive pneumococcal disease (IPD), and it is included in all licensed pediatric and adult pneumococcal vaccines (PCV10, Pneumosil, PCV13, PCV20, and PPSV23) as well as in the new vaccines under development (17). Thus, accurate identification of this serotype is paramount for disease surveillance, including the monitoring of vaccine impact. In this study, we investigate variants of serotype 14 isolated from three children in PNG who were hospitalized with an acute respiratory infection. These variants have mutations in the *cps* locus, resulting in the loss of capsule production. These belong to the Group I lineage of nonencapsulated pneumococci, consisting of pneumococci that possess either a partial or full *cps* locus but also possess mutations that lead to the inability to produce capsule (18). This group includes pneumococci with defective serotype 14 *cps* loci as well as various *cps* loci of other serotypes (10, 11, 18).

Although similar variants have been identified previously (10, 11), we have conducted an in-depth investigation which revealed that these 14-like pneumococci exhibit discrepant serotyping results, depending on the method employed. Despite the inability of these pneumococci to produce capsule, most molecular serotyping methods assessed in this study designated these isolates as serotype 14. Not only were discrepant serotyping results obtained by different methods, but the mistyping potential was also further complicated by inconsistent serotype calls of each isolate by the same method (observed for the SeroCall and qPCR methods) (Table 2). The latter was due to mutations of different length and location across the *cps* locus of the different isolates (e.g., the deletion in PMP1437 included *wzy*, the target for the qPCR method, whereas this gene is intact in PMP1438 and PMP1514).

Although overlooking mutations in the *cps* is a known limitation of most molecular methods for serotyping, the occurrence of these variants in the population raises concerns around the potential for reducing the accuracy of serotyping data used to make decisions around vaccination programs. This is particularly relevant for the variants described in the present study, as their mistyping (as serotype 14) would incorrectly classify them as a vaccine serotype, despite their ability to evade the opsonizing serotype 14 antibodies induced by vaccination (Fig. 3). Thus, although molecular methods for serotyping generally perform well, displaying a high concordance with phenotype, it is important to note their limitations, particularly in the serotyping of isolates from countries where limited information on genetic variation in the pneumococcal population is available.

All three 14-like isolates identified in this study belong to different genetic lineages (Table 1), and the mutations in their *cps* loci differ in size and location (Fig. 2). This suggests convergent evolution, where mutations have occurred on multiple occasions within independent lineages of serotype 14 pneumococci. It is plausible that capsule loss may be advantageous under specific circumstances, such as during initial adherence (19) or in reducing the metabolic burden on the bacterium.

Although nonencapsulated pneumococci rarely cause pneumonia or IPD, they can cause other diseases including conjunctivitis and otitis media (20). Additionally, nonencapsulated pneumococci play important roles in shaping the pneumococcal population. Hiller et al. (21) showed recombination between a nonencapsulated and a serotype 14 strain during a carriage episode. Interestingly, the nonencapsulated recombinant had enhanced biofilm-forming capability compared with its serotype 14 parent. Biofilm formation is important for colonization and persistence in the nasopharynx. Nonencapsulated pneumococci are more likely to undergo genetic recombination compared with capsulated strains (20). Chewapreecha et al. (22) identified higher recombination frequencies in nonencapsulated pneumococci compared with serotype 14 isolates of the same genetic background. In PNG, multiple serotype carriage is high (6), providing optimal conditions for horizontal gene transfer between co-colonizing strains. Therefore, although the 14-like pneumococci are unlikely to be directly involved in disease, they have the potential to drive genetic diversity in the PNG pneumococcal population, making these variants a concern if they are not efficiently targeted by vaccination.

Pneumococcal serotyping is essential in obtaining reliable data for disease surveillance and in monitoring vaccine impact. As this information is used by decision-makers to inform policy, the accuracy of serotyping methods is vital. Our study highlights the potential for serotype diversity and mistyping, particularly where there is limited genomic information, such as in low- and middle-income settings. Therefore, it is essential that when putative variants with divergent capsule loci are detected by molecular serotyping methods, these results are validated by phenotypic approaches. In this situation, isolates should be examined further by Quellung and/or latex agglutination, with the final serotype call being based on the phenotype. Lastly, our study highlights the need to monitor the pneumococcal population for variants that could escape vaccine-induced protection, including those in IPD and pneumonia surveillance programs.

## MATERIALS AND METHODS

### Pneumococcal identification and molecular serotyping from nasopharyngeal samples.

Cases of moderate to severe pneumonia (2 to 59 months of age) at the Eastern Highlands Provincial Hospital and community health care clinics within the Goroka town, as well as their contacts and caregivers, were recruited as part of the PneuCAPTIVE study (12). Criteria for inclusion in the study have been described previously (12). Ethical approval for this study was obtained from the PNG Institute of Medical Research Institutional Review Board (IRB no. 1510), PNG Medical Research Advisory Committee (MRAC 15.19/16.09), and The Royal Children’s Hospital Human Resources Ethics Committee (HREC reference no. 35249). Following written informed consent, nasopharyngeal swabs were collected from patients in accordance with World Health Organization guidelines (23). Swabs were placed in 1 mL skim milk, tryptone, glucose, and glycerol medium (24), then vortexed and aliquoted prior to storing at −80°C. Samples were then shipped to the Murdoch Children’s Research Institute on dry ice and stored at −80°C until use. Swabs were screened for pneumococci using qPCR, targeting the *lytA* gene (25) as described previously (26). Pneumococci from swabs were cultured on selective media (horse blood agar supplemented with 5 μg/mL gentamicin) for DNA extraction and DNA microarray to infer the serotype(s) as described previously (27), using Senti-SPv1.5 microarray slides (BUGS Bioscience).

### Bacterial isolates.

Pneumococcal isolates were purified from nasopharyngeal swabs by plating sample aliquots on horse blood agar supplemented with 5 μg/mL gentamicin. An α-hemolytic colony from each sample was purified. These isolates (PMP1437, PMP1438, and PMP1514) were confirmed as pneumococci by optochin sensitivity testing and whole-genome sequencing (multilocus sequence typing and pathogenwatch ID).

### Whole-genome sequencing and molecular serotyping.

For all molecular methods, genomic DNA was extracted from pneumococcal isolates using the QIAcube HT with the QIAamp 96 DNA QIAcube HT Kit (Qiagen) as described previously (27). Quantitative PCR was performed, targeting the serotype 14 *wzy* gene. Primer and probe sequences, concentrations, and cycling conditions were kept as described previously (16). DNA from the 14-like isolates (and the serotype 14 positive-control strain PMP829) was extracted as described above. The qPCRs were performed using the GoTaq Probe qPCR Master Mix (Promega), in which each reaction consisted of 1x master mix, 300 nM forward and reverse primer, 100 nM probe (6-FAM dye and BHQ1 quencher), and 0.5 ng/μL genomic DNA. The reactions were run under the following cycling conditions: 95°C for 10 min, then 40 cycles of 95°C for 15 sec and 60°C for 1 min. Isolates were defined as serotype 14 if a cycle threshold (ct) value less than 40 was obtained.

For whole-genome sequencing, library preparation was performed using the Illumina DNA Prep kit (Illumina) and sequenced in 2 × 150 bp paired end reads on the NovaSeq platform. For molecular serotyping, reads were run through PneumoCaT 1.2 (13), seroBA 1.0.2 (14), (recommended k-mer size of 71) or SeroCall 1.0 (15), using the default parameters.

To fully characterize the *cps* locus in the isolates from this study, long read nanopore sequencing was conducted. Genomic DNA (1 to 2 μg) was prepared using the Ligation Sequencing Kit with Native Barcoding Expansion (Oxford Nanopore Technologies), followed by sequencing on a MinION SpotON flow cell (R9.4.1) (Oxford Nanopore Technologies). Short- and long-read data were then filtered with Trim Galore v.0.6.4 (28) and qcat v.1.1.0 (Oxford Nanopore Technologies), respectively. The combining and assembly of short- and long-reads was performed with Unicycler v.0.4.8, using the default settings (29). Schematic comparisons of the 14-like capsule locus sequences to the serotype 14 reference (4) were conducted using Easyfig version 2.2.5 (30).

### Phenotypic serotyping.

Serotyping by Quellung and latex agglutination was performed against all SSI pneumococcal pools and type 14 antisera. For Quellung serotyping (31), antisera from the Statens Serum Institut (SSI) (https://ssidiagnostica.com) was used. Pneumococci were cultured on horse blood agar plates and incubated at 37°C with 5% CO_2_ overnight. A slightly turbid bacterial suspension (2 McFarland standard) was prepared from these cultures in saline. On a glass microscope slide, 1 μL of the suspension was mixed with 1 μL of the antisera of interest. The mixture was then examined under a microscope (400× magnification). A positive reaction with the antisera of interest was defined as cells with a ‘swollen’ appearance that were more visible under the microscope.

For serotyping by latex agglutination (32), latex reagents were prepared by adsorbing SSI antisera to polystyrene latex beads as described previously (33). A saline suspension of the pneumococcal culture was prepared (4 or 5 McFarland standard). On a glass microscope slide, 10 μL of the bacterial suspension was mixed with 10 μL of the latex reagent and rotated on an orbital shaker at ~140 rpm for 2 min. A positive reaction was indicated by visible agglutination and clearing of the suspension.

### Opsonophagocytosis assay.

Sera from a cohort of healthy individuals vaccinated with either one or two doses of PCV7 and/or PPSV23 from the Fiji Pneumococcal Project (FiPP) (34) were inactivated at 56°C for 30 min and serially diluted 1:2, with 10 μL placed into each well. Aliquots of each pneumococcal strain were thawed and diluted to ~5 × 10^4^ CFU/mL, and 20 μL of this suspension was added to each well and then incubated for 15 min at 37°C, 5% CO_2_ to allow opsonization to occur. Into each well, 10 μL of complement and ~4 × 10^5^ differentiated HL60 cells (a neutrophil cell line, previously washed twice with Hanks’ balanced salt solution + 0.2% BSA) were added and incubated, shaking at 37°C, at 220 rpm for 45 min to promote killing of opsonized pneumococci. After chilling on ice, 5 μL of each reaction were spotted, allowed to form drips onto Todd-Hewitt agar supplemented with 0.5% (wt/vol) yeast extract, and allowed to dry prior to the addition of an overlay with 2,3,5-triphenyltetrazolium chloride (TTC) and incubation at 37°C, 5% CO_2_ overnight. The next day, the number of pneumococcal colonies was counted and used to determine the viable count and to calculate the opsonophagocytic index (OI), defined as the interpolated dilution of serum that shows 50% of serotype-specific killing of pneumococci. The lower limit of detection in the assay is 4. The OIs of samples that do not kill 50% of bacteria were reported as 4 for analysis purposes. A positive response was defined as an OI of >4. Nonspecific killing was assessed by comparing the viable count of pneumococci incubated with complement versus heat-inactivated complement.

### Data availability.

The 14-like capsule locus sequences have been deposited in GenBank (accession nos. OM328061-OM328063).
